# Clinical outcomes of conventional surgery versus MitraClip® therapy for moderate to severe symptomatic mitral valve regurgitation in the elderly population: an institutional experience

**DOI:** 10.1186/s12872-017-0523-4

**Published:** 2017-03-20

**Authors:** Anthony Alozie, Liliya Paranskaya, Bernd Westphal, Alexander Kaminski, Mohammad Sherif, Magnus Sindt, Stephan Kische, Jochen Schubert, Doreen Diedrich, Hüseyin Ince, Gustav Steinhoff, Alper Öner

**Affiliations:** 1 Department of Cardiac Surgery, Heart Center Rostock, Schillingallee 35, 18057 Rostock, Germany; 2 Department of Cardiology, Heart Center Rostock, Ernst-Heydemannstrasse 6, 18057 Rostock, Germany; 3 Department of Anesthesiology and Intensive Care Medicine, Heart Center Rostock, Schillingallee 35, 18057 Rostock, Germany; 4 Kardiologie und Konservative Intensivmedizin, Landsbergerallee 49, 10249 Berlin, Germany; 5 Institute For Biostatistics and Informatics in Medicine And Ageing Research, Ernst Heydemanstr. 8, 18057 Rostock, Germany

**Keywords:** Mitral valve surgery, MitraClip therapy, Heart valve surgery, Mitral regurgitation

## Abstract

**Background:**

The aim of this study was to compare treatment of moderate to severe symptomatic mitral regurgitation (MR) with either conventional surgery or the mitral valve edge-to-edge device (MitraClip®) in very elderly patients. The newly introduced MitraClip device has demonstrated promising acute results in treating this patient cohort. Also noteworthy is the fact that patients who otherwise would have been denied surgery are increasingly referred for treatment with the MitraClip device. We sought to review our institutional experience, comparing outcomes in both surgical and MitraClip arms of treatment in the elderly population with symptomatic MR.

**Methods:**

From October 2008 through October 2014, 136 consecutive patients aged ≥ 80 with moderate to severe symptomatic MR were scheduled for either conventional surgery or MitraClip intervention. 56 patients ≥ 80 were operated for symptomatic MR and 80 patients ≥ 80 were treated with the mitraClip device. Patients suitable for this study were identified from our hospital database. Patients ≥80 with moderate/severe symptomatic MR treated with either conventional surgery or the MitraClip device were eligible for our analysis. We compared the surgical patient cohort with the mitraClip patient cohort after eliminating patients that did not meet our inclusion criteria. Forty-two patients were identified from the conventional cohort who were then compared with 42 patients from the mitraClip cohort. Forty-two patients (50%) underwent mitral valve repair or replacement (40.5% functional MR, 59.5% organic/mixed MR) and 42 patients (50%) underwent MitraClip intervention (50% functional MR, 50% organic/mixed MR). Associated procedures in the conventional surgical group were myocardial revascularization 38%, pulmonary vein ablation 23.8%, left atrial appendage resection 52.4% and PFO occlusion 11.9%.

**Results:**

Patients who underwent MitraClip treatment were though slightly older but the differences did not attain statistical significance (mean, 82.2 ± 1.65 vs 81.7 ± 1.35 years, *p* = 0,100), had lower LVEF (mean, 47.6 ± 14.2 vs 53.4 ± 14.3, *p* = 0.072), lower logistic EuroScore II (mean, 11.3 ± 5.63 vs 12.1 ± 10.6, *p* = 0.655) but higher STS risk score (mean, 11.8 ± 6.7 vs 8.1 ± 5.6, *p* = 0.008) respectively compared to surgical patients. Procedural success was 100% vs 96% in surgery and MitraClip groups respectively. Thirty -day mortality was 7.1% vs 4.8% (*p* = 1.000) in surgery and MitraClip group respectively. Residual postoperative MR ≥2 at discharge was present in none of the patients treated surgically, whereas this was the case in 10 (23.8%) patients treated with the MitraClip device. At 1 year a cumulative number of four (9.52%) patients died in the surgical group vs 9 (21.4%) patients who died in the MitraClip group.

**Conclusions:**

Elderly patients presenting with moderate to severe symptomatic MR may either be treated by conventional surgery or with the MitraClip device with acceptable acute outcomes. The decision for treatment with the MitraClip device should not depend on age alone rather on cumulative risk of conventional surgery. Concomitant cardiac pathologies, often times treated simultaneously during surgery for symptomatic MR may be omitted, if patients are scheduled outright to MitraClip treatment. The effect of concomitant cardiac pathologies left untreated at the time of interventional mitral valve repair on outcome after MitraClip therapy remain widely unknown.

## Background

More than 9% of patients ≥75 years have significant MR [1]. The proportion of individuals aged ≥75 with symptomatic MR is estimated to become a growing clinical problem. The decision whether to refer an elderly patient with symptomatic MR for mitral valve surgery and appropriate timing of referral is a highly debated issue. Age, severe MR and reduced left ventricular function amongst others have been identified as independent patient factors associated with non referal for mitral valve surgery [2]. Though, due to outstanding improvements in diagnostic modalities, patient selection criteria and operative techniques over the years, the operative mortality, in-hospital morbidity and long term outcomes associated with mitral valve surgery has significantly improved [3]. However, the benefit of surgery in the very elder population despite these significant improvements over the years has not been clearly documented. Increasing life expectancy and associated increase in the prevalence of valvular heart diseases, especially MR amongst the very elderly population is expected to become a rising clinical problem in the industrialized nations. Many patients at this advanced age will likely present with concomitant cardiac pathologies, necessitating surgical attention [4, 5]. The novel MitraClip device is anticipated to play a crucial role in addressing MR in this elderly patient population. In this context, staged percutaneous management of concomitant pathologies may be feasible in some cases. Coronary heart disease, aortic valve stenosis and certain pathologic rhythms may be treated in a staged total percutaneous manner. Data on the fate of moderate tricuspid regurgitation left untreated during conventional surgery remain insufficient and this is equally the case with percutaneous mitral valve repair. The impact of these residual pathologies on short and long term outcome has not been fully evaluated, leaving complete surgical intervention as gold standard [6]. Though there are indications that the novel MitraClip device may confer superior acute outcomes, with lower procedural mortality in symptomatic patients, however, long term outcomes, especially in the very elderly is not well known. The impact of residual MR, atrial septal defect, atrial fibrillation, incomplete revascularization and tricuspid valve disease in this patient population treated with the MitraClip device is not known. Despite higher mortality and morbidity in the acute phase post surgery, surgery in most cases offer complete repair of concomitant cardiac pathologies [7–10]. We sought to compare the overall outcome of conventional mitral valve surgery vs MitraClip treatment in a single center cohort of 84 patients.

### Patient selection and methods

#### Patient selection

The criteria for selecting patients eligible for MitraClip intervention has been previously described by Paranskaya et al. [11]. We at first found 80 patients who underwent mitraClip intervention. Forty-two patients were found suitable for comparing with the surgical cohort. In the surgical arm, we retrospectively selected patients ≥80 years who presented with moderate/severe symptomatic MR and were treated with conventional surgery with the aim of comparing them with a similar MitraClip treatment population.

A team of experienced cardiac surgeons, cardiologists and anesthesiologists selected patients eligible for conventional surgery and decision was based on the guidelines as stipulated by the joint task force on the management of valvular heart diseases of the European Society of Cardiology [12]. Transthoracic echocardiography (TTE) followed by transoesophageal echocardiography (TEE) examination at baseline were applied for general screening purposes. A search into our department surgical database for patients ≥ 80 who received mitral valve surgery (repair or replacement) during this time frame yielded at first 56 patients. Forty-two patients suitable for our analysis were retained after excluding patients with concomitant tricuspid-, aortic valve surgeries, mitral valve stenosis, active endocarditis of any valve or intracardiac structures and emergency settings requiring hemodynamic or respiratory support prior to surgery (Table 1 depicts concomitant procedures in the surgical group). In-hospital data were retrospectively retrieved from each patient’s personal file. Surgical patients who received concomitant myocardial revascularization were included because MitraClip patients with significant coronary stenosis received percutaneous coronary interventions prior to MitraClip therapy. All patients with justifiable EuroScore II and STS scores were referred for surgery after obtaining informed consent.

**Table 1:** Patients baseline demographics and clinical risk factors

	Surgery (*n* = 42)	MitraClip (*n* = 42)	*p*-value
Age (years), mean ± SD, (n)	81.7 ± 1.35, (42)	82.2 ± 1.65, (42)	0.100^῀^
Female gender, n (%)	23 (54.8)	18 (42.9)	0.383^*^
BMI (kg/m^2^), mean ± SD, (n)	26.3 ± 4.30, (42)	25.9 ± 3.96, (42)	0.682^῀^
Previous cardiac surgery, n (%)	7 (16.7)	7 (16.7)	1.000^*^
Coronary artery disease, n (%)	31 (73.8)	26 (61.9)	0.350^*^
COPD, n (%)	10 (23.8)	14 (33.3)	0.469^*^
Atrial fibrillation, n (%)	23 (54.8)	29 (69.0)	0.261^*^
Pulmonary hypertension, n (%)	32 (76.2)	40 (95.2)	0.026^*^
Chronic renal disease, n (%)	26 (61.9)	32 (76.2)	0.238^*^
Arterial hypertension, n (%)	36 (85.7)	39 (92.9)	0.483^*^
Diabetes mellitus, n (%)	14 (33.3)	18 (42.9)	0.501^*^
Cardiac decompensation, n (%)	25 (59.5)	37 (88.1)	0.006^*^
Logistic Euroscore II, mean ± SD, (n)	12.1 ± 10.6, (42)	11.3 ± 5.63, (42)	0.655^῀^
NYHA functional class, n (%)			
II	8 (19.0)	1 (2.4)	0.039^**^
III	27 (64.3)	30 (67.9)	
IV	7 (16.7)	11 (21.4)	
Operative Data			
Previous PCI, n (%)	-	7 (16.7)	
Concomitant CABG, n (%)	16 (38)	-	-
Concomitant pulmonary vein ablation, n (%)	10 (23.8)	-	-
Concomitant LAA resection, n (%)	22 (52.4)	-	-
Concomitant PFO occlusion, n (%)	5 (11.9)	-	-

῀ Student’s unpaired *t*-test

* Fisher’s exact Test for categorical data

** Chi-square test for categorical data

## Methods

This study is based on our experience with very elderly patients with symptomatic moderate- severe MR, who underwent correction of this valve pathology during the period between October 2008 and October 2014.

Patients underwent surgical correction of MR with either mitral valve repair, replacement or placement of ≥1 MitraClip devices. A retrospective assessment was then perfomed after all follow-up data were retrieved. This was accomplished by obtaining all medical records from nursing home personnel, family physician or patient/family members. Follow- up was obtained at different intervals post index procedure (last survey or death) in all 84 patients included in this study.

### Follow-up

All patients were followed up after discharge by the family physicians and cardiologist with regular physical examination, including evaluation of NYHA score, ECG, TTE and TEE if need be. This was at first performed during the first 3 months post treatment and then dependent on clinical status at 6 months and on yearly basis there after. Two surgical patients were lost to follow-up. All other patients discharged alive from hospital were followed up at different intervals post index procedure.

### Treatment procedures

Mitral valve surgery was performed via median sternotomy in all 42 surgical patients. Cardio pulmonal bypass was installed and valve repair or replacement with variable concomitant surgical procedures on the arrested heart under mild hypothermia (32 °C) performed. Access to mitral valve was gained either via a direct left atrial or trans septal incision depending on weather PFO occlusion was planned. Complete rigid or semi rigid annuloplasty rings or in case of valve replacement solely tissue prosthesis used. All procedures were quality controlled using intra operative TEE. MitraClip treatment was performed in the hybrid suit as previously described by *paranskaya* et al. [11].

### Statistical analysis

Statistical analysis was conducted by using SPSS statistical package 22.0 (SPSS Inc. Chicago, Illinois, USA). Descriptive statistics were computed for continuous and categorical variables. The statistics computed included mean and standard deviations (SD) of continuous variables and are presented as mean ± SD, frequencies and relative frequencies of categorical factors. Testing for differences of continuous variables between the study groups was accomplished by the *t*-test for independent samples or the Mann-Whitney *U* test, as appropriate. Test selection was based on evaluating the variables for normal distribution employing the Kolmogorov-Smirnov test. Comparisons between the study groups for categorical variables were done using the chi-square test or the Fisher’s exact test. For unbiased results the matched pairs method for the arrangement of statistical twins was used. For the matching process a correspondence of the parameters age, sex and MR Type was used. In specific, matching for age was performed using frequency matching to assure that cases and controls have similar distributions over strata defined by age. The matching on age involved the construction of three separate categories. The age categories were as follows: 80–82, 83–84 and ≥ 85 years respectively.

The period of time to death or last follow-up was estimated using the Kaplan-Meier method. Differences between curves were assessed by Mantel’s log-rank test for censored survival data.

The Cox proportional hazards model was used to assess the independence of survival from prognostic factors. Univariate analyses were performed by computation of hazard ratios, 95% - confidence intervals and examination the significance by the Wald statistic to reveal unadjusted significant associations between prognostic variables and survival. Altogether, *p*-values ≤ 0.05 were considered to be statistically significant and all reported *p*-values resulted from two-sided statistical test.

## Results

### Patient baseline characteristics and risk profile

From October 2008 through October 2014, 84 consecutive patients aged ≥ 80 with moderate to severe symptomatic MR were scheduled for either conventional mitral valve surgery or MitraClip intervention. Patient data were analysed retrospectively. All patients suitable for our analysis were identified from our hospital database. Patients ≥ 80 with symptomatic MR treated with either conventional surgical or MitraClip intervention were eligible for our analysis. Patients with concomitant tricuspid-, aortic valve surgeries, mitral valve stenosis, active endocarditis of any valve or intracardiac structures and emergency settings requiring hemodynamic or respiratory support prior to surgery were excluded from this analysis. Patient baseline characteristics and risk factors are depicted in Table 1.

Forty-two patients (50%) underwent mitral valve surgery with mitral valve repair in 30 patients (71.4%) or replacement in 12 patients (28.6%). Of the 30 patients who received mitral valve repair, minimally invasive (MIC) mitral valve repair was performed in five (16.7%). Procedural success was similar (100%) in both repair and replacement groups respectively. There was no significant differences in both groups in terms of 30 day mortality (2 vs 1 patients). 40.5% of surgically treated patients had functional MR whereas 59.5% had organic/mixed MR. Forty-two patients (50%) underwent MitraClip implantation, 50% of which had functional MR whereas 50% had organic/mixed MR respectively. Seven patients (16.7%) in each group had previous cardiac surgery in the past. Associated procedures in the conventional surgical group were myocardial revascularization (38%), pulmonary vein ablation (23.8%), left atrial auriculum resection (52.4%) and PFO/ASD-closure (11.9%). All but a few important differences between patients in both arms of our analysis attained statistical significance. Patients in the MitraClip cohort had higher STS risk score (*p* = 0.008). They were though older, but the differences did not attain statistical difference (*p* = 0.100). MitraClip patients also had lower proportion of individuals in NYHA classes II (*p* = 0.039), had higher number of individuals with previous cardiac decompensation (*p* = 0.006). Patients in the conventional surgical cohort had higher mean logistic EUROSCORE II, but the differences did not attain statistical significance (*p* = 0.655). With regards to echocardiographic parameters, patients in the MitraClip cohort had higher proportion of individuals with functional MR, however this difference did not attain a statistical significance (*p* = 0.511). Table 2 shows further echocardiographic parameters.

**Table 2:** Preoperative echocardiographic parameters

	Surgery (*n* = 42)	MitraClip (*n* = 42)	*p*-value
MR ≥ 3, n (%)	42 (100)	42 (100)	
LVEF, mean ± SD, (n)	53.4 ± 14.3, (38)	47.6 ± 14.2, (42)	0.072^῀^
LVEDD (mm), mean ± SD, (n)	55.3 ± 7.47, (30)	57.5 ± 9.54, (42)	0.296^῀^
LVESD (mm), mean ± SD, (n)	38.2 ± 6.81, (12)	44.1 ± 10.6, (42)	0.077^῀^
sPAP (mmHg), mean ± SD, (n)	47.2 ± 8.04, (13)	51.0 ± 12.2, (42)	0.212^῀^
MR Type			
Functional, n (%)	17 (40.5)	21 (50.0)	0.511^*^
Organic + mixed, n (%)	25 (59.5)	21 (50.0)	

῀ Student’s unpaired *t*-test

* Fisher’s exact Test for categorical data

### Procedural and in-hospital outcomes

Procedural success was 100% in the conventional surgery group and 95.2% in the MitraClip group (*p* =0,494). In one MitraClip patient, MR grade was three at discharge. Another patient with symptomatic MR and significant inter atrial shunt was treated with mitral valve replacement and ASD closure prior to discharge. Thirty surgical patients (71.4%) received an annuloplasty using a complete rigid or semi rigid ring. Twelve patients received tissue valve replacement (28.6%). Nine patients in the MitraClip cohort were treated with one clip device, 22 patients with two clips, eight patients with three clips and the remaining three patients with four clips devices each. In-hospital- stay was significantly increased in the surgical group compared to the MitraClip group (18.0 ± 7.60 vs 8.81 ± 6.40, *p* < 0.001) as well as incidents of acute kidney injury (10 vs 2, *p* = 0.026) and pneumonia (12 vs 3, *p* = 0.020). Details of periprocedural characteristics of all patients are depicted in Table 3.

**Table 3:** Post operative results/characteristics

	Surgery (*n* = 42)	MitraClip (*n* = 42)	*p*-value
Procedure time (min), mean ± SD, (n)	214.1 ± 57.2, (42)	186.4 ± 73.7, (41)	0.059^~^
Ventilation time ≥ 24h, n (%)	8 (19.0)	1 (2.4)	0.029^*^
ICU stay(days), mean ± SD, (n)	3.71 ± 4.00, (41)	(0)	
In Hospital stay(days), mean ± SD, (n)	18.0 ± 7.60, (41)	8.81 ± 6.40, (42)	<0.001^~^
Procedural success, n (%)	42 (100)	40 (95.2)	0.494^*^
In-hospital-death, n (%)	3 (7.1)	1 (2.4)	0.616^*^
30-day-mortality, n (%)	3 (7.1)	2 (4.8)	1.000^*^
Major bleeding, n (%)	2 (4.8)	3 (7.1)	1.000^*^
Tracheotomy, n (%)	1 (2.4)	0 (0.0)	
Acute kidney injury, n (%)	10 (23.8)	2 (4.8)	0.026^*^
Dialysis post surgery, n (%)	4 (9.8)	1 (2.4)	0.202^*^
Pneumonia, n (%)	12 (28.6)	3 (7.1)	0.020^*^
Myocardial infarction, n (%)	1 (2.4)	0 (0.0)	
Permanent Pacemaker, n (%)	9 (21.4)	0 (0.0)	
Stroke, n (%)	4 (9.5)	0 (0.0)	0.116^*^
Predischarge MR Grades, n (%)			
MR 0	34 (82.9)	6 (14.3)	<0.001^**^
MR I	7 (17.1)	26 (61.9)	
MR II	0 (0.0)	9 (21.4)	
MR III	0 (0.0)	1 (2.4)	

~ Student’s unpaired *t*-test

* Fisher’s exact Test for categorical data

** Chi-square test for categorical data

### Follow- up, mortality and morbidity

Follow-up was performed at various intervals post index procedure in both surgical and the MitraClip cohort. Mean follow-up times were 2.08 ± 1.68 years for the surgical cohort and 0.75 ± 0.50 years for the MitraClip cohort respectively for a total of 87.53 and 31.57 patient years respectively. Overall early mortality and morbidity rates are shown in Table 3. Three patients (7.1%) in the mitral surgical cohort died within 30 days of the index procedure. At 1 year this number increased to four (9.5%). In the MitraClip cohort, two patients (4.8) died within 30 days of the index procedure and this number increased to nine (21.42%) at 1 year. Univariate analysis revealed that the only predictor of 30-day mortality was COPD in both the MitraClip and surgical cohort (HR 7.26, 95% CI 0.76–69.9, *p* = 0.086). None of the other preoperative variables was significantly associated with this outcome variable (Table 4). Ventilation time > 24 h was more frequent in the surgical group. ICU and in-hospital stays (18.0 ± 7.60 vs 8.81 ± 6.40, *p* < 0.001) were longer in the surgical cohort. In-hospital-death (3 vs 1, *p* = 0.616), acute kidney injury (10 vs 2, *p* = 0.026), need for dialysis (4 vs 1, *p* = 0.202), pneumonia (12 vs 3, *p* = 0.02) and need for permanent pacemaker implantation (9 vs 0) were more frequent in the surgical group (Table 3).

**Table 4:** Predictors of 30- day Mortality in octogenarians treated with MVS vs. MC

	univariate Cox Regression								
	all Patients (*n* = 84)			MVS (*n* = 42)			MC (*n* = 42)		
Variable	Hazard Ratio	95%-CI	*p*-value	Hazard Ratio	95%-CI	*p*-value	Hazard Ratio	95%-CI	*p*-value
Logistic Euroscore									
> 10 vs. <=10^a^	**	**	**	**	**	**	**	**	**
NYHA baseline									
class 3/4 vs. class 1/2^a^	**	**	**	**	**	**	**	**	**
Baseline LVEF									
> 50% vs. <=50%^a^	1.07	0.07–17.1	0.963	**	**	**	1.18	0.07–18.9	0.905
Cardiac decompensation									
yes vs. no^a^	**	**	**	**	**	**	**	**	**
Age									
80–82 years vs. 83–85 years^a^	**	**	**	**	**	**	**	**	**
Sex									
female vs. male^a^	1.11	0.16–7.85	0.920	**	**	**	**	**	**
Coronary artery disease									
yes vs. no^a^	1.44	0.15–13.8	0.753	**	**	**	0.60	0.04–9.53	0.714
Diabetes mellitus									
no vs. yes^a^	1.82	0.19–17.5	0.604	**	**	**	0.77	0.05–12.3	0.851
COPD									
yes vs. no^a^	7.26	0.76–69.9	0.086	2.85	0.18–45.6	0.459	**	**	**
GFR ≤ 30									
yes vs. no^a^	**	**	**	**	**	**	**	**	**
GFR 30–50									
yes vs. no^a^	1.77	0.25–12.5	0.570	**	**	**	**	**	**
Atrial fibrillation									
yes vs. no^a^	1.68	0.17–16.1	0.655	0.71	0.04–11.4	0.810	**	**	**
Pulmonary hypertension									
yes vs. no^a^	**	**	**	**	**	**	**	**	**

**not determinable, because there are only censored times

^a^indicates reference category

### Echocardiographic outcomes

Predischarge MR 0, I° and II° were found in 34, seven and zero patients versus six, 26 and nine patients in the mitral surgical and MitraClip groups respectively (*p* < 001, Table 3). At follow up nine patients in the mitral surgical group had MR I°, one patient had MR II°. In the Mitraclip cohort, three patients remained at MR I°, 23 patients had MR I°, nine patients had MR II°, three patients had MR III°. One Patient had mitral stenosis II° post intervention in the Mitraclip cohort. In the surgical cohort, no mitral stenosis was recorded. In general, surgery was more effective in reducing the grade of MR acutely compared to MitraClip.

## Discussion

Mitral valve surgery in the elderly oftentimes multi morbid population is a highly debated issue. Although more elderly patients with MR are increasingly being identified and referred for surgery, still as much as 50% are often denied surgery due to perceived higher risk and lower benefit expectation from surgical correction of MR [2]. This tendency is partly justifiable for secondary MR due to the fact that results of surgery are unpredictable in improving patient outcomes. Moreover surgery for secondary MR has never been compared with guidelines directed medical therapy (GDMT) alone for left ventricular dysfunction in a prospective randomized manner. Studies comparing outcomes of patients with secondary MR treated with mitral valve repair and those treated with GDMT are mostly retrospective in nature, using propensity score matching and have failed to demonstrate an advantage of surgery in improving survival [13]. However, results of surgical elimination of MR in primary (degenerative) MR on the other hand, especially reconstructive surgery are excellent.

**Fig. 1:**
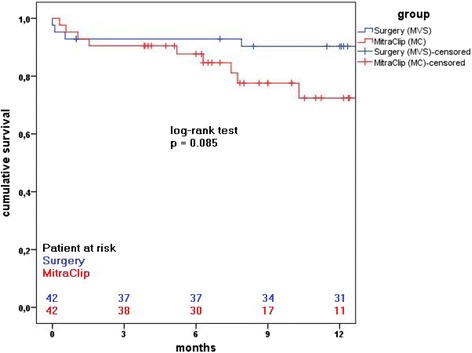
Kaplan-Meier survival rate after MitraClip and surgery

Some studies provided insight into potential poorer prognosis for patients with functional MR and left ventricular dysfunction and this is especially true for patients with recent acute myocardial infarction and for patients with chronic ischemic and non ischemic cardiomyopathy [14]. It was shown that even with the current GDMT, there was always a tendency to adverse prognosis due to untreated ischemic functional MR. Therefore this category of patients who remain symptomatic despite GDMT and CRT may be a reasonable target for surgical or interventional therapy of functional MR. Swaans et al. recently reported mortality rates in patients receiving neither surgery nor percutaneous therapy for MR that even exceeded that of either surgical or interventional therapy [15]. This report provided new insights into the potential survival benefits of mitral valve intervention (both surgical and interventional) compared with GDMT alone in patients with LV dysfunction and secondary MR [15]. In the Survival and Ventricular Enlargement Trial (SAVE) patients with even mild functional MR after myocardial infarction were more likely to experience cardiovascular mortality (29% vs. 12%; *p* < 0.001), and severe heart failure (24% vs. 16%; *p* < 0.0153) than those without MR [16]. Trichon et al. reported 2057 patients with ischemic MR and chronic LV systolic dysfunction and showed residual MR to be an independent predictor of mortality. They also found significantly lower survival rates at 1, 3 and 5 years of heart failure patients with moderate to severe MR versus those without MR or with mild MR [17]. Hence, none referral of elderly symptomatic patients with MR for at least surgical evaluation, due to age alone is highly debatable. The optimal timing for surgery or percutaneous intervention is also critical in reducing mortality rates associated with both surgery and interventional therapy. The percutaneous edge-to-edge device has since its first application in human, continuously improved, and currently demonstrates superb acute interventional outcomes in the elderly population with MR classified as high surgical risk [18, 19]. Though operative risks of mitral valve surgery reduced significantly during the last decades [20], however quality of life after such surgical interventions, a factor of great importance in the elderly population has rarely been reported. Maissano et al [21] found that quality of life was suboptimal in more than half of elderly patients following mitral valve surgery and that surgical technique did not impact quality of life at follow up. They identified several factors significantly related to increased Minnesota Living With Heart Failure (MLHF) - score at follow-up after valve surgery in the elderly. Age, preoperative atrial fibrillation (or rhythm other than sinus rhythm at follow up), EuroSCORE, Charlson score and recurrent/residual MR at follow up amongst others were significantly associated with poorer quality of life at follow up. They suggested that quality of life should be a better indicator of successful surgery/intervention in this age group than survival alone. In our study, though the 30-day mortality was slightly higher in the surgical cohort, but there was equalization of mortality rates as time progressed, with even higher mortality rate in the MitraClip group (depicted in Fig. 1). At 1- year- follow up surgical patients had lower MR recurrence than found in the Mitraclip group. Kaneko et al in their recent study found that residual MR 2+ was associated with poorer prognosis in patients with impaired LV function, renal dysfunction, and severe heart failure. They suggested that the optimal endpoint of MC procedure should be individualized according to each patient's baseline characteristic [22].

The impact of tricuspid regurgitation left untreated at time of Mitraclip intervention was recently reported by ohno et al [23] who found that estimates for freedom from combined endpoint of death and rehospitalisation for heart failure were worse in moderate/severe tricuspid regurgitation group of patients treated with the MitraClip device compared with those with none/mild tricuspid regurgitation (67.7% vs 88.8% log-rank *p* = 0.015). Overall NYHA was also found to be worse in this group of patients with moderate/severe tricuspid regurgitation, despite improved LVEF and ventricular volumes at 12 month-follow-up. Since the current practice of Mitraclip intervention does not include concomitant cardiac interventions in the aortic valve, tricuspid valve, atrial fibrillation ablation or complete myocardial revascularisation, all of which have high prevalence in the elderly population, adequate patient selection should identify patients who would rather benefit from complete surgical intervention despite age.

Mitral valve surgical procedures in the very elderly with symptomatic MR is associated with profound operative morbidity and mortality rates, especially in deem of frequently associated comorbidities [10]. Mirabel et al [2] from the Euro Heart Survey on heart valve diseases reported that in a group of 396 patients with symptomatic MR, surgery was denied in 49%, with age, impaired LVEF and several comorbidities playing the main role in the decision not to offer surgery. However untreated symptomatic secondary MR seems to confer even higher mortality and morbidity. MitraClip therapy, though has acceptable procedural outcomes, but long term results are pending and the durability of acute reduction of MR in certain patients remains controversial. De Bonis et al. recently failed to confirm the results of EVEREST II trial in which it was observed that the MItraClip results remained stable in the long term if initial therapy was successfull. Infact they found that patients with FMR, despite optimal initial results of MitraClip intervention had the tendency to develop recurrent MR. Whether FMR at this time can be seen as risk factor for recurrent MR after MitraClip therapy remain to be confirmed in a prospective randomized trial.

Treatment of concomitant cardiac pathologies, which have increased prevalence in this age group, is very limited. The impact of untreated concomitant cardiac pathologies, left untreated at the time of MitraClip therapy versus complete surgical interventions on the mid and long term outcomes should be the subject of future studies. Our acute procedural outcomes in the mitral surgical group is in line with published data on mortality and morbidity rates after mitral valve surgery in the very elderly, despite several concomitant surgical procedures and high EuroScore II values, and mid term outcomes are impressive.

As the number of elderly patients with mitral valve disease is expected to increase rapidly, there is need for data providing evidence on the benefits and harm of both conventional MV surgery and several percutaneous therapeutic options, being propagated for MR treatment in these high risk patients. As percutaneous mitral valve repair options are rapidly gaining ground as alternative option for mitral valve repair in these high risk surgical patients, at the same time some patients are reoperated successfully after failed interventions. However this is expected to decrease with perfection of this technology. Adequate patient selection prior to decision for either conventional surgery or MitraClip therapy should be pursued in the future. Age alone should at present not be a criterium for pro MitraClip therapy, rather comorbidities predisposing to high prohibitive risk of surgery and in those cases with ambiguous survival benefit of surgery. Benefit of surgery for MR due to ischemic cardiomyopathy remains a highly debated issue [24–26]. Multidisciplinary team approach is key to providing optimal individually tailored treatment for elderly patients with symptomatic MR. Primary and secondary MR are very different entities in terms of etiology, management and prognosis and these differences should be put into account during multidisciplinary decision pro or contra surgery or interventional treatment of symptomatic MR.

## Study limitations

This is a retrospective, single-center observational study with a very limited number of observed patients. There may be some, unidentified confounding factors that might have influenced certain results, so strong conclusions from our study should not yet be deduced or generalized, until our results is confirmed by prospective and randomized clinical trials. Follow-up data were obtained by both facsimile questionnaire and telephone interviews with patients family practitioner and cardiologist. Hence, accuracy of grading NYHA functional class, MR grades may have been compromised by interobserver bias. Patient selection for the respective procedures did not follow standard operating procedures, but rather decisions were made after interdisciplinary consultation in each case within a heart team. The completely different burden of both treatment arms, with patients in the surgical arm receiving different and at times multiple concomitant procedures with subsequent impact on early clinical outcome is another important limitation.

In terms of quality of life, unfortunately due to the retrospective nature of our study, quality of life was not assessed preoperatively and therefore assessing quality of life during follow-up would not provide data usefull for any reasonable comparison.

Another important limitation is that the number of patients whose data were available for 1-year followed-up was less in the mitraClip group.

## Conclusions

Elderly patients presenting with moderate to severe symptomatic MR may either be treated by conventional surgery or with the MitraClip device with acceptable acute outcomes. The decision for treatment with the MitraClip device should not depend on age alone rather on cumulative risk of conventional surgery. Concomitant cardiac pathologies, often times treated simultaneously during surgery for symptomatic MR may be omitted, if patients are scheduled outright to MitraClip treatment. The effect of concomitant cardiac pathologies left untreated at the time of interventional mitral valve repair on outcome after MitraClip therapy remain widely unknown.

## References

[1] Nkomo VT, Gardin JM, Skelton TN, Gottdiener JS, Scott CG, Enriquez-Sarano M (2006). Burden of valvular heart diseases: a population-based study. Lancet.

[2] Mirabel M, Iung B, Baron G, Messika-Zeitoun D, Détaint D, Vanoverschelde JL, Butchart EG, Ravaud P, Vahanian A (2007). What are the characteristics of patients with severe, symptomatic, mitral regurgitation who are denied surgery?. Eur Heart J.

[3] Detaint D, Sundt TM, Nkomo VT, Scott CG, Tajik AJ, Schaff HV, Enriquez-Sarano M (2006). Surgical correction of mitral regurgitation in the elderly: outcomes and recent improvements. Circulation.

[4] Eurostat. Population structure and ageing. Available at: http://ec.europa.eu/eurostat/statistics-explained/index.php/Population_structure_and_ageing. Accessed 15 Mar 2017.

[5] Alfieri O, Maisano F (2011). Mitral valve surgery in the elderly: New insights and unanswered questions. Eur Heart J.

[6] Paranskaya L, D’Ancona G, Bozdag-Turan I, Akin I, Kische S, Turan GR, Rehders T, Ortak J, Nienaber CA, Ince H (2013). Residual mitral valve regurgitation after percutaneous mitral valve repair with the MitraClip® system is a risk factor for adverse one-year outcome. Catheter Cardiovasc Interv.

[7] Neuhold S, Huelsmann M, Pernicka E, Graf A, Bonderman D, Adlbrecht C, Binder T, Maurer G, Pacher R, Mascherbauer J (2013). Impact of tricuspid regurgitation on survival in patients with chronic heart failure: unexpected findings of a long-term observational study. Eur Heart J.

[8] Alozie A, Westphal B, Kische S, Kaminski A, Paranskaya L, Bozdag-Turan I, Ortak J, Schubert J, Steinhoff G, Ince H (2013). Surgical revision after percutaneous mitral valve repair by edge-to-edge device: when the strategy fails in the highest risk surgical population. Eur J Cardiothorac Surg.

[9] Vahanian A, Baumgartner H, Bax J, Butchart E, Dion R, Filippatos G (2007). Task Force on the Management of Valvular Hearth Disease of the European Society of Cardiology; ESC Committee for Practice Guidelines. Guidelines on the management of valvular heart disease: the Task Force on the Management of Valvular Heart Disease of the European Society of Cardiology. Eur Heart J.

[10] Biancari F, Schifano P, Pighi M, Vasques F, Juvonen T, Vinco GJ (2013). Pooled estimates of immediate and late outcome of mitral valve surgery in octogenarians: a meta-analysis and meta-regression. Cardiothorac Vasc Anesth.

[11] Paranskaya L, D’Ancona G, Bozdag-Turan I, Akin I, Kische S, Turan GR, Divchev D, Rehders T, Westphal B, Schubert J, Nienaber CA, Ince H (2013). Percutaneous mitral valve repair with the MitraClip system: perioperative and 1-year follow-up results using standard or multiple clipping strategy. Catheter Cardiovasc Interv.

[12] Vahanian A, Alfieri O, Andreotti F, Antunes MJ, Barón-Esquivias G, Baumgartner H, Borger MA, Carrel TP, De Bonis M, Evangelista A, Falk V, Lung B, Lancellotti P, Pierard L, Price S, Schäfers HJ, Schuler G, Stepinska J, Swedberg K, Takkenberg J, Von Oppell UO, Windecker S, Zamorano JL, Zembala M (2012). Guidelines on the management of valvular heart disease (version 2012): the Joint Task Force on the Management of Valvular Heart Disease of the European Society of Cardiology (ESC) and the European Association for Cardio-Thoracic Surgery (EACTS);.ESC Committee for Practice Guidelines (CPG); Joint Task Force on the Management of Valvular Heart Disease of the European Society of Cardiology (ESC); European Association for Cardio-Thoracic Surgery (EACTS). Eur J Cardiothorac Surg.

[13] Wu AH, Aaronson KD, Bolling SF, Pagani FD, Welch K, Koelling TM (2005). Impact of mitral valve annuloplasty on mortality risk in patients with mitral regurgitation and left ventricular systolic dysfunction. J Am Coll Cardiol.

[14] Benjamin MM, Smith RL, Grayburn PA (2014). Ischemic and functional mitral regurgitation in heart failure: natural history and treatment. Curr Cardiol Rep.

[15] Swaans MJ, Bakker AL, Alipour A, Post MC, Kelder JC, de Kroon TL, Eefting FD, Rensing BJ, Van der Heyden JA (2014). Survival of transcatheter Mitral valve repair compared with surgical and conservative treatment in high-surgical-risk patients. JACC Cardiovasc Interv.

[16] Lamas GA, Mitchell GF, Flaker GC, Smith SC, Gersh BJ, Basta L, Moyé L, Braunwald E, Pfeffer MA (1997). Clinical significance of mitral regurgitation after acute myocardial infarction. Survival and ventricular enlargement investigators. Circulation.

[17] Trichon BH, Felker GM, Shaw LK, Cabell CH, O’Connor CM (2003). Relation of frequency and severity of mitral regurgitation to survival among patients with left ventricular systolic dysfunction and heart failure. Am J Cardiol.

[18] Glower DD, Kar S, Trento A, Lim DS, Bajwa T, Quesada R, Whitlow PL, Rinaldi MJ, Grayburn P, Mack MJ, Mauri L, McCarthy PM, Feldman T (2014). Percutaneous mitral valve repair for mitral regurgitation in high-risk patients: results of the EVEREST II study. J Am Coll Cardiol.

[19] Taramasso M, Maisano F, Denti P, Latib A, La Canna G, Colombo A, Alfieri O. Percutaneous edge-to-edge repair in high-risk and elderly patients with degenerative mitral regurgitation: Midterm outcomes in a single-center experience. J Thorac Cardiovasc Surg. 2014. doi: 10.1016/j.jtcvs.2014.03.036. [Epub ahead of print]. 10.1016/j.jtcvs.2014.03.036 24768099

[20] Gazoni LM, Kern JA, Swenson BR, Dent JM, Smith PW, Mulloy DP, Reece TB, Fedoruk LM, Lisle TC, Peeler BB, Kron IL. A change in perspective: results for ischemic mitral valve repair are similar to mitral valve repair for degenerative disease. Ann Thorac Surg. 2007;84:750–7. 10.1016/j.athoracsur.2007.04.098 17720371

[21] Maisano F, Viganò G, Calabrese C, Taramasso M, Denti P, Blasio A, Guidotti A, Alfieri O (2009). Quality of life of elderly patients following valve surgery for chronic organic mitral regurgitation. Eur J Cardiothorac Surg.

[22] Kaneko H, Neuss M, Weissenborn J, Butter C. Impact of residual mitral regurgitation after MitraClip implantation. Int J Cardiol. 2017;227:813–819. 10.1016/j.ijcard.2016.10.054 27823895

[23] Ohno Y, Attizzani GF, Capodanno D, Cannata S, Dipasqua F, Immé S, Barbanti M, Ministeri M, Caggegi A, Pistritto AM, Chiarandà M, Ronsivalle G, Giaquinta S, Farruggio S, Mangiafico S, Scandura S, Tamburino C, Capranzano P, Grasso C (2014). Association of tricuspid regurgitation with clinical and echocardiographic outcomes after percutaneous mitral valve repair with the MitraClip System: 30-day and 12-month follow-up from the GRASP Registry. Eur Heart J Cardiovasc Imaging.

[24] De Bonis M, Lapenna E, Buzzatti N, La Canna G, Denti P, Pappalardo F, Schiavi D, Pozzoli A, Cioni M, Di Giannuario G, Alfieri O (2016). Optimal results immediately after MitraClip therapy or surgical edge-to-edge repair for functional mitral regurgitation: are they really stable at 4 years?. Eur J Cardiothorac Surg.

[25] Crabtree TD, Bailey MS, Moon MR, Munfakh N, Pasque MK, Lawton JS, Moazami N, Aubuchon KA, Al-Dadah AS, Damiano RJ (2008). Recurrent mitral regurgitation and risk factors for early and late mortality after mitral valve repair for functional ischemic mitral regurgitation. Ann Thorac Surg.

[26] Mihaljevic T, Lam BK, Rajeswaran J, Takagaki M, Lauer MS, Gillinov AM, Blackstone EH, Lytle BW (2007). Impact of mitral valve annuloplasty combined with revascularization in patients with functional ischemic mitral regurgitation. J Am Coll Cardiol.

